# The Awareness-First Theory: A Coherence Principle Underlying Active Inference and Physical Law

**DOI:** 10.3390/e28030306

**Published:** 2026-03-09

**Authors:** Jason Clarke

**Affiliations:** School of Human and Social Sciences, University of West London, Ealing, London W5 5RF, UK; jason.clarke@uwl.ac.uk

**Keywords:** active inference, free energy principle, phenomenal consciousness, variational principles, transcendental conditions

## Abstract

The Free Energy Principle (FEP) and Active Inference provide a unifying variational framework for modelling perception, action, learning, and self-organisation across biological systems. While highly successful at explaining how systems maintain organisation under uncertainty, these frameworks remain explicitly neutral with respect to a foundational question: why there is experience at all. This paper argues that this limitation reflects not an empirical gap but a misplaced starting point. The Awareness-First Theory (AFT) inverts the usual explanatory order by beginning from the givenness of awareness itself and asking what must be the case for any world to appear coherently. This requirement is formalised as a Coherence Principle, expressed as a variational stationarity condition, δA=0, which specifies the invariance of coherent awareness across changing appearances. I argue that familiar variational principles-most notably free-energy minimisation (δF=0) and stationary-action physics (δS=0)-can be understood as restricted projections of this parent constraint under specific abstractions. Active Inference therefore does not generate awareness but describes how locally bounded systems maintain coherence within awareness under uncertainty. Making this projection structure explicit dissolves the explanatory gap between physical process and phenomenal presence, revealing the gap itself as a category error. Although the Coherence Principle itself is transcendental rather than empirical, the AFT generates testable consequences at the level of its projections, including predicted dissociations between inferential optimisation and phenomenological coherence in dreaming, altered states, meditation, and psychopathology.

## 1. Introduction

The Free Energy Principle (FEP) and its operationalisation through Active Inference have emerged as among the most ambitious unifying frameworks in contemporary cognitive neuroscience. Together, they provide a principled account of perception, action, learning, and self-organisation across biological scales, from single cells to complex organisms [1,2,3]. Under this framework, any system that persists in a fluctuating environment must minimise variational free energy with respect to an internal generative model, thereby maintaining its integrity under uncertainty. Organisms are thus understood as self-evidencing systems: processes that continuously update beliefs about the causes of their sensory states in order to resist entropy and preserve organisation [4]. Importantly, this account explains how behaviour can appear purposive without invoking teleology, grounding agency in lawful inference rather than design.

The reach of this single variational framework has expanded rapidly into consciousness science. Dreaming has been modelled as inference under relaxed sensory precision, accounting for coherent but internally generated narratives during REM sleep [5]. Affective experience has been framed in terms of interoceptive inference, where affective valence reflects success or failure in maintaining homeostatic regulation [6]. Hierarchical self-modelling approaches locate awareness within recursive inferential loops that include the system’s own generative model [7]. Architectural proposals such as the Inner Screen reinterpret Markov blankets as nested holographic surfaces through which precision-weighted covert actions regulate global ignition [8]. More broadly, Markovian Monism treats bounded systems as admitting dual but inseparable geometries—physical and informational—thereby licensing intentional description without positing ontologically distinct substances [9]. Collectively, these developments have furnished consciousness research with a shared mathematical scaffold and a coherent mechanistic vocabulary.

Yet despite this remarkable unification, a foundational question remains unresolved. However complete the inferential account, nothing in it entails the existence of experience itself. The Free Energy Principle explains how systems behave and maintain organisation; it does not explain why there is something it is like to instantiate those dynamics [10,11,12,13]. Leading proponents have been explicit on this point: FEP is a theory of self-organisation, access, and control, not a theory of phenomenal presence [14,15] Attempts to supplement the framework via reflexive representations, dual-aspect descriptions, or higher-order inference risk redescribing structural features of cognition without addressing the givenness of experience. In short, the mathematics secures coherence of behaviour, but not the fact that there is coherent appearing at all.

Crucially, the difficulty exposed here is not empirical but structural. Standard scientific explanations begin from within an already-given world: they presuppose a space of states, lawful dynamics, and a distinction between system and environment. Within such an explanatory frame, awareness can only appear—if at all—as a contingent outcome of complexity. But if awareness is absent from the explanatory primitives, no amount of functional refinement can logically entail its presence. The “explanatory gap” thus reflects not a missing mechanism, but a misidentification of explanatory starting point [10,11,12,13].

The present paper makes this starting point explicit. Rather than introducing a new mechanism or ontological posit, the Awareness-First Theory (AFT) begins from what every scientific inquiry already presupposes: the fact that something appears at all. This fact is not inferred, hypothesised, or observed; it is given. Any attempt to deny it is self-defeating, since denial itself is an occurrence within awareness—a point recognised from Descartes’ cogito to Husserl’s “principle of all principles,” and in traditions that treat awareness as intrinsically self-revealing [16,17,18]. On this account, awareness is not an entity added to an otherwise non-experiential world, but the field within which any world can appear coherently at all.

Philosophically, this orientation stands in continuity with William James’s radical empiricism, Whitehead’s process philosophy, and phenomenological accounts of givenness, all of which treat experience as primary rather than derivative [19,20,21,22,23,24]. James argued that “pure experience” is the neutral field from which distinctions between subject and object are subsequently abstracted, and that the relations binding experience into a unity are themselves primitive [19]. These traditions articulated a powerful intuition—that nature is not composed of non-experiential material to which awareness must later be added; however, they lacked a precise formal expression compatible with contemporary theoretical science.

The AFT supplies such an expression by making explicit a structural constraint already implicit in variational science. Formally, awareness is represented as a differentiable field whose admissible transformations are constrained by a Coherence Principle, expressed schematically as a stationarity condition:

δA=0.

Here, A does not denote a substance or object, but the structured field of appearing itself. The condition δA=0 expresses the invariance of coherent presence—unity, temporal continuity, and reflexive availability—across changing appearances. This principle is not an empirical law but a transcendental constraint: it cannot be violated without undermining the possibility of observation, theory, or explanation. When made explicit, it reveals that familiar variational principles operate as restricted projections of a more general requirement of coherence. In particular,

$$\delta A=0\;\Rightarrow \;\{\begin{array}{ll} \delta F=0 & \left(\text{free-energy stationarity/active inference}\right)\\ \delta S=0 & \left(\text{stationary-action physics}\right) \end{array}$$

where the implication is structural rather than causal. Free-energy minimisation and least-action dynamics appear as lawful expressions of coherence under specific constraints and abstractions.

Technically, it is important to distinguish the quantities to which these variational principles apply. In both Active Inference and physics, stationarity is expressed via a principle of least action, but the relevant actions are defined over distinct functionals. In Active Inference, the action corresponds to a path integral of variational free energy, defined over trajectories of probabilistic beliefs parameterised by a system’s states. In physics, by contrast, the action is a path integral over a Lagrangian defined on physical state variables. Free energy itself is not minimised directly; rather, it is the action functional constructed from free energy that is rendered stationary. On the present view, these distinct variational principles operate over a shared underlying state space, but arise as different projections of a more general coherence constraint under specific abstractions.

Within this framework, bounded self-organising systems—organisms or agents—correspond to locally stabilised regions of the awareness field, individuated by Markov blankets. Their inferential activity does not generate awareness; it is the means by which local coherence is maintained under uncertainty. Structured qualitative domains—such as colour, affect, and temporal flow—are related to differentiations of this field via lawful mappings, yielding systematic correspondences between phenomenological structure and neural information geometry. Under ordinary conditions, phenomenological coherence and inferential success coincide; under altered regimes—dreaming, illusion, psychedelic states, meditation, or psychopathology—they may dissociate, offering empirically tractable tests.

The remainder of the paper proceeds as follows. Section 2 clarifies the ontological scope and limits of Active Inference. Section 3 introduces the Coherence Principle δA=0 and explicates its phenomenological and transcendental status. Section 4 develops the resulting variational hierarchy linking coherence, inference, and physical law, including a formal toy model illustrating projection structure. Section 5 shows how this framework dissolves the traditional explanatory gap. Section 6 outlines empirical implications for cognitive neuroscience and clinical research. Section 7 situates the AFT relative to existing philosophical frameworks, including Markovian Monism, and addresses potential objections. Section 8 discusses directions for mathematical development, and Section 9 concludes.

## 2. Active Inference and Its Ontological Limits

Active Inference begins from a formally simple but generative premise: any system that persists over time in a changing environment must maintain a non-equilibrium steady state. This persistence requires the system to regulate its internal dynamics so as to minimise the divergence between its expectations and the states it encounters. The Free Energy Principle formalises this requirement as the minimisation of variational free energy with respect to an internal generative model [1,2,3]. Perception, action, learning, and attention are thereby unified as aspects of a single inferential process through which a system maintains its organisation under uncertainty [4].

Within this framework, living systems are understood as self-evidencing processes. By continuously updating probabilistic beliefs about the causes of their sensory inputs, such systems act so as to confirm their own model of the world, thereby resisting entropy and preserving identity [4]. Markov blankets play a central role in this account, defining statistical boundaries that separate internal from external states while allowing their coupling via sensory and active exchanges. Neural activity, bodily action, and environmental engagement are thus not independent faculties, but complementary expressions of a single gradient flow on variational free energy.

Crucially, the explanatory power of Active Inference lies in its ability to account for purposive behaviour without invoking teleology. Apparent goal-directedness emerges from the lawful dynamics of inference rather than from design or intention. In this respect, the Free Energy Principle provides one of the most general and parsimonious accounts of biological organisation currently available.

### 2.1. Active-Inference Approaches to Consciousness

Building on this foundation, a growing body of work has extended Active Inference into the domain of consciousness science. These approaches differ in emphasis, but they share a commitment to explaining conscious phenomena in terms of inferential structure, precision control, and hierarchical organisation.

Hierarchical self-modelling accounts locate awareness within recursive inferential loops that include the system’s own generative model as an object of inference. The Beautiful Loop framework proposes that conscious awareness arises when a system not only infers the causes of its sensory states, but also models and updates its own predictive activity, closing an epistemic loop in which the model includes itself [7]. Here, awareness corresponds to a form of inferential closure: the moment at which the system’s predictions become globally available within its own modelling hierarchy.

Other approaches emphasise architectural features of the inferential process. The Inner Screen framework reinterprets Markov blankets as nested holographic surfaces on which information is inscribed and read out [8]. Conscious contents are proposed to arise on the deepest of these surfaces, where covert actions—neuromodulatory adjustments of precision—determine which information is globally broadcast. Ascending neuromodulatory systems are thereby linked to the control of conscious access, connecting the mathematics of Active Inference to identifiable neural circuits of arousal, attention, and ignition.

Further work has sought to clarify the relevant correlates of consciousness within an inferential framework. Rather than treating consciousness as tied to particular neural regions, computational correlates approaches identify it with patterns of inferential flow through belief space [15]. Here, what matters are not static structures but trajectories of belief updating and precision modulation. Consciousness corresponds to specific forms of causal organisation within these trajectories, formalised as conditions under which the causal structure of physical processes mirrors the causal structure of inferred states.

The Integrated World Modelling Theory (IWMT) combines Active Inference with insights from Global Neuronal Workspace Theory and Integrated Information Theory [25]. Consciousness is identified with the experience of modelling an integrated world, including the modelling system itself, across multiple temporal and spatial scales. Oscillatory dynamics synchronise distributed neural populations into coherent world-models, yielding unified experiential content.

Affective approaches emphasise the role of interoceptive inference. In this framework, consciousness originates not in perception or cognition but in the organism’s capacity to register the success or failure of its own regulation [6]. Affective valence reflects predicted changes in uncertainty: pleasure accompanies expected reductions in surprise, while unpleasure accompanies unpredicted increases [6]. Higher forms of awareness are elaborations of this primary affective field.

Finally, Whyte and colleagues [26] extract an explicit minimal theory of consciousness implicit in Active Inference itself. They argue that existing models converge on a shared structure in which conscious access occurs at the interface between continuous perceptual inference and discrete policy selection. Conscious contents correspond to posterior beliefs available for counterfactual evaluation and action. This formulation yields clear empirical predictions while remaining neutral on the metaphysical status of phenomenal experience.

### 2.2. Markovian Monism and Ontological Neutrality

A particularly interesting synthesis is Markovian Monism, which formalises a dual-geometry description of self-organising systems under the Free Energy Principle [9]. Any Markov-blanketed system may be described either in physical coordinates-flows of matter and energy-or in information-geometric coordinates-flows of probabilistic belief and precision. These descriptions are not ontologically distinct; they are related by a change in coordinates within a single underlying dynamic.

This framework provides a rigorous route beyond Cartesian dualism. Apparent distinctions between mind and matter arise from shifts in descriptive perspective rather than from differences in substance. Consciousness, in this framework, is a graded property of systems whose generative models possess sufficient depth, temporal extension, and self-referential structure.

However, Markovian Monism remains explicitly neutral with respect to the existence of awareness itself. While it explains how physical and informational descriptions relate, it does not specify why either description should be accompanied by phenomenal presence. Awareness is neither denied nor derived; it is bracketed. This neutrality is deliberate and principled, reflecting the scope of the framework.

### 2.3. The Remaining Limit

Despite their explanatory reach, inference-based accounts share a common limitation. They explain how systems maintain organisation, regulate behaviour, and model themselves as agents, but they do not explain why any of these processes should be accompanied by experience. The mathematics of Active Inference specifies structure, function, and dynamics; it does not entail presence.

This is not a limitation of the Free Energy Principle. It is a consequence of its explanatory stance. Active Inference begins from a third-person ontology in which phenomenality is unmentioned. Within such a framework, awareness can only appear-if at all-as an emergent correlate of inferential structure. But if awareness is absent from the explanatory primitives, no degree of refinement can logically entail its presence. The explanatory gap thus reflects a structural limitation rather than an empirical one.

Moreover, the standard physicalist assumption that experience is generated by a mind-independent world introduces an untestable posit. All observation and inference already occur within awareness; the hypothesised external cause of experience is, by definition, inaccessible. As long noted in phenomenology, scientific objectivity is achieved by abstraction from the subject, but the act of abstraction itself remains grounded in the field of experience [23,24].

Those accustomed to objectivist or neuro-reductionist frameworks may find the starting point of the AFT an unusual one. Indeed it is, but the aim here is not to deny neurocognitive mechanisms, nor to treat awareness as an empirical posit competing with physical explanation. Rather, the aim is to make explicit a constraint that all such mechanisms already presuppose: that their operations occur within a coherent field of appearing. The argument that follows proceeds structurally rather than causally. It should therefore be read as a reordering of explanatory priority, not as a replacement of empirical or mechanistic accounts. Active Inference and physical law remain indispensable; what changes is the level at which their conditions of intelligibility are addressed.

The present paper does not reject Active Inference or the Free Energy Principle. On the contrary, it treats them as among the most successful explanatory frameworks in contemporary science. The claim advanced here is that their success rests on a deeper constraint that remains implicit. Making that constraint explicit-by recognising coherence of awareness as a condition of intelligibility-does not compete with inference-based explanations, but generalises them.

In the following section, this constraint is introduced formally as the Coherence Principle δA=0, and its phenomenological and transcendental status is clarified.

## 3. The Coherence Principle (δA=0)

### 3.1. Phenomenological Motivation

Across all possible experiences-perceptual, cognitive, emotional, or reflective-the contents of awareness change, but the fact of awareness does not. Sensations arise and pass, thoughts shift, affect fluctuates, and attention moves; yet throughout these transformations there remains a continuous field within which they appear. This continuity is not inferred from theory or observation. It is directly given in experience itself. Any attempt to deny it already presupposes it, since denial is itself an occurrence within awareness [16,17,19].

This invariance concerns neither a particular object nor a representational state, but the coherence of appearing as such. Experiences are given as unified rather than fragmented, temporally continuous rather than episodic, and available rather than absent. These features persist across diverse experiential regimes, including ordinary wakefulness, dreaming, meditative absorption, and states of minimal or reduced content. Even when specific contents fade, the presence of a coherent field remains.

Importantly, this observation is not an empirical generalisation. It does not arise from sampling many experiences and inferring a common property. Rather, it is disclosed immediately in any act of awareness. One cannot step outside awareness to test whether awareness is present; its presence is the condition under which any testing, theorising, or doubting occurs. For this reason, the invariance of awareness has long been recognised in phenomenology as a datum rather than a hypothesis [17,19], and in other philosophical traditions as self-revealing or self-luminous.

This phenomenological datum motivates the Coherence Principle.

### 3.2. Formal Statement

The principle is expressed schematically as a variational stationarity condition:

δA=0.

Here, A denotes the structured field of awareness understood not as an object, substance, or inner representation, but as the field of coherent appearing itself. The symbol δ denotes admissible variation. The condition δA=0 therefore states that real configurations of awareness are those in which coherence is preserved under transformation, in the sense that intelligibility requires the maintenance of unity, temporal continuity, and reflexive availability across change. This is not a law of identity (‘awareness = awareness’) or a tautological claim of self-sameness, but a constraint on admissible transformations: it specifies the conditions under which change remains intelligible as experience at all.

This formulation does not assert that awareness is static or unchanging. On the contrary, awareness differentiates continuously into diverse contents and structures. What remains invariant is the coherence of those differentiations: unity rather than disintegration, temporal continuity rather than interruption, and reflexive availability rather than absence. The Coherence Principle thus constrains *how* awareness may change, not *whether* it may change.

The use of variational notation is deliberate. In physics and inference, variational principles do not specify particular mechanisms but define global constraints on admissible trajectories. Similarly, δA=0 does not describe a causal process occurring within awareness. It specifies the condition under which any process can appear as intelligible at all.

### 3.3. Transcendental Status

Unlike empirical principles, the Coherence Principle cannot be violated without undermining the possibility of observation, theory, or explanation. A physical law may fail under extreme conditions; an inferential model may break down in pathology or noise. By contrast, a breakdown of coherence of awareness would not constitute a counterexample to the principle, but the collapse of the conditions under which counterexamples could be recognised.

For this reason, δA=0 functions as a transcendental constraint rather than a causal law. It specifies what must be the case for any empirical domain, scientific model, or explanatory practice to be possible. In this respect, it plays a role analogous to Kantian conditions of possibility, while differing in one crucial respect: it is not posited as a feature of an unknowable subject or faculty, but is read directly off experience itself as the invariance of appearing.

This distinction matters. The Coherence Principle does not introduce a metaphysical entity beyond the reach of science. It makes explicit a constraint that science already presupposes in practice. Every measurement, model, and explanation assumes a coherent field in which results can appear, be compared, and be revised. The principle articulates this assumption rather than adding to it.

### 3.4. Relation to Scientific Variational Principles

The formal similarity between δA=0 and other variational principles is not coincidental. In physics, the principle of stationary action (δS=0) selects physically realised trajectories [27,28]. In Active Inference, free-energy stationarity (δF=0) selects inferential trajectories that maintain a system’s organisation under uncertainty [1,2]. In each case, stationarity expresses preservation of structure under constraint.

The AFT proposes that these principles are not independent laws operating at the same explanatory level, but restricted expressions of a more general requirement. The Coherence Principle specifies the most general invariance-coherence of appearing from which more specific stationarities arise when additional constraints and abstractions are imposed.

When coherence is constrained by uncertainty, embodiment, and persistence, it manifests as inferential optimisation, yielding δF=0. When phenomenological structure and reflexivity are abstracted away, coherence manifests as physical law, yielding δS=0. These relationships are structural rather than causal: the Coherence Principle does not cause inference or physics, but defines the conditions under which inferential and physical descriptions are possible.

### 3.5. Summary of the Coherence Principle

This section has introduced the Coherence Principle as a formal expression of a phenomenologically given invariant. The principle articulates the minimal condition under which anything can appear coherently at all. By making this condition explicit, the AFT reorders explanatory priority: awareness is not explained by inference or physical law; inference and physical law are explained as lawful differentiations of awareness under constraint.

The next section develops this claim by articulating a variational hierarchy linking coherence, inference, and physics, and by introducing a simple toy model that makes the logic of projection explicit.

## 4. A Variational Hierarchy: From Coherence to Inference and Physics

The central structural claim of the AFT is that the variational principles governing inference and physical dynamics are not independent laws operating at the same explanatory level, but constrained projections of a more general requirement: the preservation of coherence of awareness. This section develops that claim by articulating a variational hierarchy in which the Coherence Principle (δA=0) functions as a parent constraint, from which free-energy stationarity (δF=0) and stationary action (δS=0) arise under specific conditions and abstractions.

### 4.1. Coherence as the Most General Variational Constraint

Variational principles specify admissible trajectories by selecting those that preserve some invariant quantity under transformation [27,28]. In physics, this invariant is action; in Active Inference, it is variational free energy. In the AFT, the invariant is coherence of awareness itself.

From the perspective of Active Inference, phenomenological coherence at the first-person level is typically accompanied by measurable forms of coordination at the third-person level. When a system maintains a coherent grip on its world, this is ordinarily reflected in increased mutual information and synchrony between internal states and the structured regularities of its environment. From a first-person perspective, such coherence is lived as a stable, unified, and temporally continuous field of experience; from a third-person perspective, it may be described in terms of coordinated neural dynamics, precision-weighted coupling, or synchronisation between sensory signals and neural activity (e.g., electrophysiological measures). Importantly, these descriptions track correlated expressions of coherence rather than constituting it: phenomenological coherence and neural synchrony typically co-occur, but they are not identical, and may come apart under specific conditions.

The Coherence Principle does not compete with other variational principles, nor does it replace them. It specifies the condition under which any such principle can operate meaningfully.

At the most general level, δA=0 expresses the requirement that transformations of awareness preserve unity, temporal continuity, and reflexive availability. These features are not contingent properties of particular experiences but conditions of intelligibility. Any trajectory that destroyed them would not count as a trajectory of experience at all. Coherence is therefore the minimal constraint governing all admissible variations.

Importantly, this constraint is neutral with respect to specific contents, representations, or mechanisms. It does not privilege perception over cognition, affect over belief, or neural processes over bodily or environmental dynamics. It applies equally to all domains in which something appears coherently.

### 4.2. From Global Coherence to Local Inference: δF=0

When coherence is constrained by uncertainty, embodiment, and persistence over time, it manifests locally as inferential optimisation. A bounded region of the awareness field-individuated by a Markov blanket-must regulate the flow of sensory and active states in order to remain coherent relative to its environment. Under these conditions, preservation of coherence takes the form of approximately minimising expected surprise, formalised as variational free energy.

Technically, both physics and Active Inference express stationarity in terms of an action functional, but the action is defined over different quantities. In physics, action is a path integral of a Lagrangian over physical state trajectories and may be related, under probabilistic descriptions, to the accumulation of surprisal. In Active Inference, by contrast, the action is a path integral of variational free energy defined over trajectories of probabilistic beliefs, where variational free energy constitutes an upper bound on surprisal. This distinction reflects the presence of a Markov blanket, which separates internal inferential states from external states and requires optimisation to proceed via beliefs about hidden causes rather than direct access to physical variables. Under this view, physical action and inferential action are formally analogous variational constructions operating at different descriptive levels within a shared hierarchy.

From this perspective, free-energy minimisation does not generate awareness. It is one way in which awareness maintains local coherence under constraint. Inferential dynamics specify how a region of awareness stabilises itself by constructing and updating a generative model that mediates its coupling to the world. Neural activity, action selection, and precision control are therefore mechanisms of coherence maintenance, not sources of phenomenal presence.

This reinterpretation preserves the full empirical content of Active Inference. All standard results concerning perception, learning, attention, and action remain intact. What changes is their ontological placement. Inference is no longer tasked with explaining why experience exists, but with explaining how coherent experience is locally sustained.

### 4.3. From Coherence Under Abstraction to Physical Law: δS=0

A further abstraction yields the variational principles of physics. When phenomenological structure and reflexivity are formally abstracted away—when coherence is considered independently of how it is experienced—it appears as lawful physical dynamics. In this regime, trajectories are selected by the stationarity of action rather than by inferential optimisation.

The principle of stationary action (δS=0) thus emerges as coherence viewed under maximal abstraction. Physical laws describe how coherence propagates when only relational structure is retained and experiential givenness is bracketed. This explains why physical laws are stable, symmetric, and mathematically tractable, while remaining silent on phenomenology. Physics describes coherence stripped of its mode of appearance.

This projection-based understanding is consonant with relational and operational approaches to physics, in which physical laws are treated as constraints on relations between measurements rather than as descriptions of observer-independent properties [29].

This account does not reduce physics to phenomenology, nor does it deny the autonomy of physical explanation. Rather, it situates physical law within a broader hierarchy of description. Physical dynamics are one legitimate and indispensable way of describing coherence, but they are not ontologically self-sufficient.

### 4.4. Structural, Not Causal, Priority

The relationship between these variational principles is not causal. The Coherence Principle does not cause free-energy minimisation, nor does free-energy minimisation cause stationary action. Instead, the relationship is one of structural containment. Each principle operates within a restricted domain defined by the constraints imposed on coherence.

This distinction is crucial. Causal explanations operate within a domain of appearing phenomena; the Coherence Principle specifies the condition under which any such domain exists. To treat δA=0 as a causal law would therefore be a category mistake. Its priority is logical and structural, not temporal or mechanistic.

### 4.5. A Toy Model of Invariant Generators and Variational Projections

To make the projection structure described above explicit, it is useful to introduce a simple toy model. The purpose of this model is not to describe neural dynamics, physical processes, or phenomenology directly, but to illustrate a general mathematical point: a single invariant structure may admit multiple lawful projections, none of which contains the invariant itself.

Consider Euler’s identity:

$$e^{i\theta }=\cos\theta +i\sin\theta .$$

This identity expresses a unit-magnitude complex exponential as a rotation on the complex plane. The invariant quantity here is the modulus, $|e^{i\theta }|=1$, which remains constant under variation of θ. The real and imaginary components, cosθ and sinθ, are lawful projections of this invariant rotation onto orthogonal axes. Each projection varies smoothly and deterministically with θ, yet neither projection contains the rotational invariance itself.

Several features of this construction are instructive.

First, the invariant magnitude is not recoverable from either projection alone. Observing cosθ or sinθ provides access to a lawful trace of the rotation, but not to the generating invariance as such. No amount of refinement of either projection yields the invariant directly; it is only defined at the level of the generating structure. Even where invariant structure can be reconstructed from multiple projections by assuming an underlying symmetry group, such reconstruction presupposes the generating domain; it does not derive the existence of that domain from within any single projection.

Second, the projections are mutually consistent without being reducible to one another. The cosine and sine components are linked by a shared generator, yet they are not derivable from each other in isolation. Their coherence is explained by their common origin, not by mutual containment.

Third, the projections obey their own stationarity properties. The rotational invariance of the generator induces periodic structure in each projection, which may be described independently in variational terms. The invariant governs the space of admissible trajectories, while the projections specify particular paths within that space.

This abstract structure mirrors the variational hierarchy proposed in the AFT. The Coherence Principle (δA=0) plays the role of the invariant generator: it specifies the condition under which coherent appearing is preserved across transformation. Free-energy stationarity (δF=0) and stationary action (δS=0) correspond to distinct projections of this invariant under different constraints and abstractions.

Free-energy minimisation describes coherence projected into probabilistic belief space under conditions of uncertainty and persistence. Stationary action describes coherence projected into physical state space under abstraction from phenomenological structure. Like the sine and cosine components of Euler’s identity, these projections are lawful, mathematically tractable, and empirically indispensable, yet none of them contains the generating invariant itself.

The toy model thus clarifies a central claim of the AFT: the absence of awareness from third-person scientific descriptions does not indicate its non-existence, but reflects the nature of projection. Awareness, understood as coherence of appearing, is not something that can appear *within* its projections, just as rotational invariance does not appear as a term in its Cartesian components. What appears are structured traces-behavioural, inferential, or physical-whose coherence is governed by a deeper invariance (see Appendix A).

A further implication of the Euler construction now becomes clear. In the model, perturbations of one projection—for example, modulation of the cosine component—do not abolish the invariant modulus; they alter only a particular coordinate expression of the underlying rotation. The invariant continues to govern the admissible space of trajectories even where one projection becomes unstable or locally degraded. By structural analogy, degradation of inferential optimisation (δF≠0 locally) need not entail collapse of global coherence (δA=0). The Coherence Principle constrains the domain within which inferential and physical projections unfold, but is not identical to either. This makes intelligible, at the level of formal structure, the possibility that inferential dynamics and phenomenological coherence may diverge under specific conditions, a possibility explored empirically in Section 6. The toy model thus clarifies not only why awareness does not appear within its projections, but also why perturbations within a projection do not exhaust the invariance that governs it.

### 4.6. Local Linearity and the Structure of Lived Temporality

A further feature of the Euler construction is worth noting. Although the invariant generator is globally rotational, any smooth curve is locally indistinguishable from a straight line. At a point

$$p=e^{i\theta_{0}},$$

the first derivative

$$\frac{d}{d\theta }e^{i\theta }=ie^{i\theta }$$

defines a tangent vector that captures the instantaneous direction of change. Curvature enters only at second order. Thus, while the generating structure is circular, its local expression is linear flow. This distinction mirrors a fundamental feature of lived temporality. Experience does not present itself as circular recurrence or global closure; it presents as continuous unfolding. The invariant structure constrains the whole trajectory, but what is given at any moment is the local direction of change.

This local structure also clarifies the phenomenological thickness of the present. In Husserlian terms, the primal impression corresponds to the point of contact p; retention corresponds to the infinitesimal arc immediately preceding it; and protention corresponds to the tangent direction along which the curve unfolds. The global closure of the circle is not experienced directly, just as rotational invariance does not appear within its Cartesian projections. What is lived is a first-order neighbourhood of the invariant structure: a continuous, directed flow that preserves coherence without revealing its generating symmetry. In this way, the Euler model not only illustrates projection structure but also shows how invariant coherence can manifest as temporally extended presence without reintroducing awareness as an object within its own projections.

### 4.7. Summary of the Variational Hierarchy

This section has articulated a variational hierarchy in which the Coherence Principle (δA=0) functions as a parent constraint. Under local constraint and uncertainty, it appears as free-energy stationarity (δF=0); under abstraction from phenomenological structure, it appears as stationary action (δS=0). The Euler toy model makes this projection structure explicit, showing how an invariant can govern multiple lawful descriptions without being contained in any of them.

In the next section, this structural insight is used to dissolve the traditional explanatory gap between physical process and phenomenal experience.

## 5. Dissolving the Explanatory Gap

The “explanatory gap” in consciousness science can be framed as the problem of explaining how subjective experience arises from non-experiential physical processes [10,11,12,13]. Within this framing, physical descriptions appear complete with respect to structure and function, yet silent on why there is something it is like to instantiate those structures. This silence has been taken to indicate an ontological incompleteness: a missing ingredient, property, or psychophysical mechanism required to bridge the gap between matter and mind.

The AFT dissolves this problem by showing that the gap arises from a structural misinterpretation rather than an explanatory failure. The error consists of treating third-person descriptions-physical or inferential-as if they were intended to contain the conditions of their own appearance. Once the logic of projection is made explicit, this demand is revealed as incoherent.

As shown in Section 4, variational principles such as δF=0 and δS=0 are lawful projections of a more general invariant: coherence of awareness. These projections preserve structure under constraint, but they do not and cannot contain the generating invariant itself. The absence of awareness from third-person science is therefore not anomalous; it is structurally required. To expect awareness to appear within physical or inferential descriptions is to commit a category error analogous to expecting rotational invariance to appear as a term within its Cartesian projections.

On this account, the explanatory gap does not mark a place where science has failed to explain something that exists. It marks a place where explanation has been misapplied. Third-person science explains relations, dynamics, and regularities within the field of appearance. It does not, and cannot, explain the existence of that field itself, because the field is the condition under which explanation occurs. Awareness is not an effect produced by physical processes; physical processes are structured appearances within awareness.

This reframing resolves a longstanding dilemma. If one assumes that the physical world is ontologically fundamental, then experience appears as an inexplicable add-on, leading either to dualism or to eliminativism. If one instead assumes that experience is fundamental, physical law appears threatened or derivative. The AFT avoids this forced choice by distinguishing between generators and projections. Awareness is fundamental not as a substance, but as an invariant condition of coherence. Physical and inferential laws remain fully real, fully objective, and fully explanatory within their domains, while no longer bearing an impossible explanatory burden.

Importantly, this dissolution does not rest on denying empirical correlations between brain processes and experience. Neural dynamics, bodily action, and environmental coupling remain indispensable for understanding how particular experiences are structured, modulated, and disrupted. What changes is the direction of explanation.

This distinction also clarifies why attempts to “solve” the hard problem by adding representational reflexivity or higher-order states inevitably fall short [12,30]. Such approaches enrich the structure of projection but leave the generator untouched. No increase in complexity at the level of projection can yield the invariant from which projection itself derives.

The explanatory gap thus dissolves once the correct explanatory roles are assigned. Awareness does not need to be derived from physics or inference because it is not something that comes into being within them. It is the invariant field within which physical and inferential descriptions operate. What appeared as a metaphysical mystery is revealed instead as a misunderstanding of explanatory scope.

In this sense, the AFT does not compete with existing scientific frameworks. It explains why they work as well as they do, and why they stop where they must. The gap between objective description and subjective presence is not a flaw in science, but a signature of projection. Recognising this transforms the hard problem from an intractable puzzle into a resolved misunderstanding.

In the next section, the empirical implications of this reordering are considered. While the Coherence Principle itself is not an empirical hypothesis, its projections generate testable consequences for neuroscience, psychology, and clinical science-particularly in regimes where inferential optimisation and phenomenological coherence come apart.

## 6. Empirical Implications and Testable Consequences

The Coherence Principle δA=0 is not itself an empirical hypothesis. As a transcendental constraint, it specifies a condition of possibility for empirical inquiry rather than a claim within it. Nevertheless, the AFT yields clear and testable consequences at the level of its projections. These consequences concern the relationship between phenomenological coherence and inferential optimisation, and they bear directly on ongoing research in cognitive neuroscience, psychiatry, and consciousness science.

### 6.1. Dissociation Regimes: Coherence Without Optimisation

Standard Active Inference models implicitly assume that phenomenological coherence tracks inferential success. Under typical waking conditions, this assumption is well supported: coherent experience coincides with effective free-energy minimisation, accurate prediction, and adaptive action. The AFT predicts, however, that this alignment is contingent rather than necessary.

Specifically, the AFT predicts the existence of regimes in which inferential optimisation degrades locally (δF≠0) while global phenomenological coherence remains intact (δA=0). In such regimes, experience does not collapse into incoherence but reorganises along alternative coherence-preserving trajectories. Several well-studied phenomena already instantiate this pattern.

Dreaming provides a canonical case. During REM sleep, sensory precision is attenuated and action is suppressed, yet experience remains temporally continuous, unified, and richly structured [5]. From the perspective of the AFT, this reflects a shift in the projection constraints governing coherence: inferential success relative to the external environment is suspended, but coherence within awareness is preserved through internally generated dynamics.

Psychedelic states exhibit a related but distinct dissociation. Under serotonergic psychedelics, precision hierarchies are disrupted, leading to increased entropy in belief updating and reduced top-down constraint [31]. Despite this, subjects frequently report heightened experiential intensity, unity, and salience. The AFT predicts precisely this pattern: local inferential instability accompanied by global coherence maintained at the level of awareness, often experienced as expanded or “unbounded” presence.

Certain meditative practices provide a third dissociation regime. In states of minimal or contentless awareness, representational activity and goal-directed inference are markedly reduced, yet awareness remains vivid, stable, and continuous [32,33]. These states pose a challenge for any account that ties awareness to representational complexity, but they follow naturally from the AFT, in which minimal differentiation corresponds to a maximally symmetric coherence state.

### 6.2. Psychopathology and Fragmentation of Coherence

The AFT also reframes psychopathological phenomena. Conditions such as psychosis, severe dissociation, and depersonalisation are modelled within Active Inference as failures of precision weighting, belief updating, or generative modelling [34,35]. While these descriptions remain valid, the AFT adds an additional dimension: the integrity of global phenomenological coherence itself.

Psychosis, as seen through this framework, is not merely inferential error but a fragmentation of coherence across scales. Hallucinations and delusions correspond to locally coherence-preserving trajectories that fail to integrate with broader constraints, leading to experiential disunity. Importantly, this predicts that phenomenological disruption should not map one-to-one onto inferential failure: patients may retain pockets of intense experiential coherence even as global integration deteriorates.

Similarly, dissociative disorders can be understood as protective coherence-preserving responses under extreme uncertainty, in which integration is selectively suspended to maintain local stability. This reframing suggests that therapeutic interventions should be evaluated not only in terms of inferential accuracy or symptom reduction, but in terms of their effects on experiential coherence [36,37].

### 6.3. Neural and Computational Implications

At the neural level, the AFT predicts that markers of phenomenological coherence will not always coincide with markers of optimal inference. Measures such as neural entropy, signal diversity, or prediction error may increase even as subjective coherence persists or intensifies. Conversely, highly optimised inferential dynamics may coexist with diminished experiential richness, as in certain automatised or dissociative states.

This motivates a distinction between neural correlates of inferential optimisation and neural correlates of coherence preservation. While these can overlap, the AFT predicts systematic divergences under specific conditions. Identifying such divergences offers a concrete empirical programme: to dissociate neural signatures of δF=0 from those associated with stable phenomenological presence.

Computationally, the AFT suggests that models of consciousness should not aim to “generate” awareness but to model the conditions under which coherence is locally maintained, modulated, or disrupted. This reframes model validation: success is not measured by whether a model produces conscious-like behaviour, but by whether it correctly predicts patterns of alignment and dissociation between inference and experience.

### 6.4. Clinical and Experimental Predictions

Several testable predictions follow. First, interventions that alter precision weighting without disrupting global coherence should produce experiential changes without loss of presence. Second, states characterised by minimal inference but preserved awareness should be systematically accessible and stable under appropriate conditions. Third, clinical improvements should correlate more strongly with restored coherence than with mere inferential accuracy.

These predictions are compatible with existing Active Inference frameworks but extend them by introducing coherence as an explicit explanatory dimension. Importantly, they do not require new measurement tools or speculative constructs. They require only a re-interpretation of existing data within a clarified variational hierarchy.

In summary, while the Coherence Principle itself is not falsifiable, its projections are: systematic failures of the predicted dissociations between inferential optimisation and phenomenological coherence would count against the framework. The AFT thus preserves empirical accountability while correcting the ontological interpretation of what empirical results can and cannot explain. In the next section, the theory is situated relative to existing philosophical frameworks and potential objections are addressed.

## 7. Relation to Existing Philosophical Frameworks

It is important to note that several objections may be raised against the orientation developed here. Some will object to the transcendental starting point itself, arguing that science should restrict itself to third-person, empirically tractable primitives; others will question whether phenomenological givenness warrants ontological priority, or whether formal variational notation is appropriate outside established physical domains. One may object that awareness is accessible only subjectively and therefore cannot serve as an epistemic foundation. The AFT does not deny this subjectivity; rather, it treats it as precisely what cannot be eliminated without circularity, since objectivity itself is constituted through structured modes of appearing. Still others may worry that the AFT collapses into idealism, panpsychism, or an unfalsifiable metaphysical stance. These concerns are legitimate points of philosophical disagreement, but they do not bear on the internal logic of the framework as presented. The AFT does not deny the autonomy of empirical science, nor does it seek to replace inferential or physical explanations; rather, it clarifies their scope by making explicit the conditions under which they are intelligible at all. While the Coherence Principle itself functions as a transcendental constraint rather than an empirical hypothesis, the framework is empirically vulnerable at the level of its projections, as outlined in Section 6. Objections that target the premises of the theory therefore concern starting points and explanatory priorities, not testable consequences or internal consistency. In what follows, the aim is not to resolve these philosophical disputes exhaustively, but to situate the AFT clearly within the landscape they define.

The AFT does not emerge in a philosophical vacuum. Its central claims resonate with long-standing traditions in metaphysics and phenomenology, while also diverging from them in decisive ways. This section clarifies those relations, both to situate the AFT historically and to make explicit what is genuinely novel about its contribution.

### 7.1. Parmenidean Invariance and the Primacy of Being

The claim that coherence of awareness is invariant across all change bears a clear structural affinity to the ontology of pre-Socratic philosopher, Parmenides, for whom what-is (to eon) is ungenerated, imperishable, and invariant beneath all appearance. On a superficial reading, Parmenides appears to deny change altogether. On a deeper reading, however, his argument concerns the impossibility of non-being: whatever appears must, in some sense, be—a point already implicit in his claim that “the same is for thinking and for being” [38,39].

The AFT can be understood as a contemporary reformulation of this insight. Change is not denied, but reinterpreted as lawful differentiation within an invariant field of coherence. The Coherence Principle δA=0 plays the role of Parmenidean being-not as a static substance, but as a stationarity condition governing admissible transformations. What persists is not any particular content, but the coherence of appearing itself.

Unlike Eleatic monism, the AFT does not collapse multiplicity into illusion. Instead, it provides a variational account of how multiplicity is compatible with invariance. In this respect, it can be seen as completing, rather than contradicting, the Eleatic intuition.

### 7.2. Kantian Transcendental Conditions

The AFT also aligns closely with the transcendental project initiated by Kant, whose central insight was that certain conditions-space, time, and the categories-are not derived from experience but are conditions for the possibility of experience. These conditions are not empirical laws but structural constraints that make empirical knowledge possible at all [40].

The Coherence Principle occupies a similar logical role. Like Kant’s forms of intuition, δA=0 is not something that could be discovered or refuted empirically, because any empirical discovery already presupposes it. Where the AFT departs from Kant is in its scope and formalisation. Rather than multiple a priori forms imposed by a subject, the AFT posits a single invariant constraint governing the field of appearance itself, without appeal to a transcendental ego.

In this sense, the AFT can be seen as a post-Kantian transcendental theory: it retains the insight that explanation has preconditions, while eliminating the need for a subject-object bifurcation at the foundational level.

### 7.3. Phenomenology and Givenness

The phenomenological tradition—from Husserl onwards—recognised that experience is given prior to theoretical abstraction, and that scientific objectivity arises through a bracketing of this givenness rather than its elimination—a position closely related to William James’s radical empiricism, which treats “pure experience” as the fundamental field from which distinctions between subject and object are subsequently abstracted [19]. Husserl’s “principle of all principles” holds that whatever presents itself in intuition is to be accepted as it presents itself [17].

The AFT inherits this commitment to givenness but departs from phenomenology in one crucial respect. Classical phenomenology describes the structure of experience but typically refrains from formalisation, treating mathematical abstraction with suspicion. The AFT instead treats phenomenological invariance as something that can-and must-be expressed formally if it is to integrate with contemporary science.

In this way, the AFT translates the central insights of phenomenology into a variational language continuous with physics and neuroscience. The awareness field is not an object of reflection but the formalisation of what phenomenology has always pointed to: the unity and continuity of appearing.

### 7.4. Projective Reflexivity and Functional-Geometry Approaches

Recent work has proposed that phenomenal stability and unity may be formally characterised in terms of projective reflexivity within multiscale functional geometries of the brain. In particular, Poznanski [41] developed a self-intending projection framework in which invariant constraints are enacted through recursive functional organisation across neural scales. Such approaches provide an important mathematical account of how coherence-like properties may be realised within biological systems. The AFT is compatible with these accounts at the level of projection: functional brain geometries can be understood as one way in which coherence-preserving constraints are locally enacted under biological and inferential conditions. However, the AFT differs in explanatory starting point. Where projective-reflexive approaches treat functional geometry as primary, the AFT treats such geometries as derivative descriptions—lawful projections of a more general transcendental constraint that is not exhausted by its neural implementation. In this sense, projective reflexivity characterises how coherence is stabilised within specific systems, while the Coherence Principle specifies the condition under which any such stabilisation is intelligible at all.

### 7.5. Relation to Panpsychism and Neutral Monism

The AFT can be superficially compared to panpsychism, but the resemblance is limited. Panpsychism distributes experience across fundamental physical constituents, typically by positing proto-phenomenal properties of matter [42]. The AFT does not ascribe experience to particles, fields, or physical objects. Instead, it treats physical objects themselves as structured appearances within awareness.

Similarly, while the AFT shares affinities with neutral monism-particularly in its refusal to privilege either mind or matter as ontologically fundamental-it departs by identifying a specific invariant (coherence of awareness) rather than an unspecified neutral “stuff” [43]. The neutrality in the AFT is not between mind and matter, but between perspectives: first-person and third-person descriptions are treated as projections of the same underlying coherence.

### 7.6. Completing Markovian Monism

The closest contemporary analogue to the AFT within theoretical neuroscience is Markovian Monism [9]. Markovian Monism argues that systems can be described equivalently in physical or informational coordinates, with no need for ontological dualism. The AFT endorses this dual-geometry insight but argues that it remains incomplete.

Without an explicit account of why there is something it is like to occupy either geometry, Markovian Monism remains ontologically neutral with respect to awareness. The AFT completes this picture by identifying coherence of awareness as the invariant that makes both geometries intelligible in the first place. Physical and informational descriptions are not merely dual aspects of the same system; they are coordinated projections of a deeper coherence condition.

In this sense, the AFT does not oppose Markovian Monism but grounds it. The monism is preserved, but its ontological centre is relocated from abstract system dynamics to the condition of appearance itself.

### 7.7. Summary of Philosophical Position

The AFT occupies a distinctive position in the philosophical landscape. It affirms with Parmenides, the primacy of invariance; with Kant, the necessity of transcendental conditions; with phenomenology, the givenness of experience; with contemporary neuroscience, the indispensability of variational principles.

Its novelty lies in unifying these strands within a single formal constraint, expressed in a language continuous with modern theoretical science. By doing so, the AFT avoids both metaphysical inflation and explanatory deflation. It neither adds mysterious entities nor eliminates what is most evident.

The next section turns from philosophical positioning to mathematical development, outlining how the awareness field, the Coherence Principle, and their projections may be rigorously formalised in future work.

## 8. Mathematical Development and Future Work

The present paper has prioritised conceptual clarity and ontological structure over full mathematical formalisation. This choice is deliberate. The AFT introduces a shift in explanatory priority, and such shifts must be made intelligible before they are formalised. Nevertheless, the framework is designed from the outset to admit rigorous mathematical development, and several avenues for such development are already clear.

The core mathematical task is to express the awareness field A and its Coherence Principle δA=0 within an explicit variational and geometric framework. The guiding intuition is that awareness can be treated as a differentiable manifold [44] whose admissible trajectories are those that preserve phenomenological coherence. In this setting, the Coherence Principle functions as a stationarity condition on a functional defined over A, analogous in form-but not in interpretation-to the action functionals of physics or the free-energy functionals of inference.

A natural starting point is differential geometry. One may treat A as a smooth manifold equipped with a metric structure that encodes relations of phenomenological similarity and integration. Variations in this structure that preserve global coherence then define geodesic or extremal paths in the awareness manifold. Under appropriate constraints, projections of these paths yield the familiar geometries of physical spacetime and information geometry. In this sense, physical and inferential manifolds appear as quotient or projection spaces derived from A, rather than as ontologically primitive arenas.

Information geometry provides a second, closely related route. Active Inference already relies on the geometry of probability distributions, particularly the Fisher information metric and associated gradient flows [2,45]. The AFT suggests extending this formalism by treating informational geometries as local charts on the awareness manifold, valid under specific coarse-grainings. One can envisage a Qualia Mapping Function as a smooth map from regions of A to structured phenomenological manifolds, providing a principled way to relate qualitative structure to neural and informational geometry. A full formal definition of such a mapping—and the conditions under which it is well-defined, smooth, and empirically constrained—lies beyond the scope of the present paper and is deferred to future work.

Gauge-theoretic formulations offer a further promising direction [29]. Coherence preservation can be interpreted as invariance under certain transformations of representational frame, suggesting that phenomenological unity may correspond to a gauge symmetry of the awareness field. Here, breakdowns of coherence—whether in psychopathology or altered states—would correspond to symmetry breaking or changes in gauge fixing, providing a principled way to connect experiential fragmentation with dynamical instability.

Finally, the relation between δA=0, δF=0, and δS=0 invites formal treatment using category theory or variational hierarchies [40]. One may seek to define a functorial relationship between the awareness manifold and its projections, clarifying in precise terms how constraints, coarse-grainings, and abstractions yield the laws of inference and physics as limiting cases. Such a framework would make explicit the sense in which the AFT “completes” existing variational theories without replacing them.

These mathematical developments are ongoing and will be presented in future work. Their success or failure will not determine the validity of the central claim advanced here, which is transcendental rather than empirical. What they will determine is the extent to which the AFT can be integrated into the existing formal apparatus of theoretical neuroscience and physics. The present paper aims to establish the conceptual groundwork for that integration, making clear both the necessity of the coherence principle and the form its formalisation is likely to take.

## 9. Conclusions

This paper has argued for a minimal but fundamental reordering of explanatory priority in cognitive neuroscience and consciousness research. Rather than attempting to derive experience from physical or inferential processes, the Awareness-First Theory (AFT) begins from the one condition that every explanation already presupposes: the coherent givenness of awareness itself. From this starting point, the familiar variational principles of inference and physics are recovered not as generators of experience, but as lawful projections of a deeper coherence constraint.

The central proposal is the Coherence Principle, expressed as the stationarity condition δA=0. This principle does not function as an empirical law, nor as a speculative metaphysical postulate. It specifies a transcendental constraint: the condition under which anything can appear as a world at all. Once this constraint is made explicit, the long-standing explanatory gap between physical process and phenomenal presence is revealed to be a category error. Third-person descriptions explain relations and dynamics *within* awareness; they cannot, in principle, explain the existence of awareness itself.

By situating Active Inference and least-action physics as structured projections of this parent variational principle, the AFT preserves the full empirical power of existing frameworks while clarifying their ontological scope. Inference does not create coherence; it maintains it locally under uncertainty. Physical law does not ground experience; it describes stable regularities abstracted from it. This reordering neither diminishes neuroscience nor inflates phenomenology. It assigns each its proper explanatory role.

Importantly, this move is conservative rather than revisionary. No new entities are introduced, no empirical results are denied, and no speculative mechanisms are proposed. What changes is the logical centre from which explanation proceeds. Awareness is not added to the picture; it is recognised as the field within which the picture already appears.

The empirical implications of this shift are non-trivial. By distinguishing coherence from inferential optimisation, the AFT predicts systematic dissociations already hinted at in dreaming, altered states, meditation, and psychopathology. These predictions do not compete with Active Inference models but extend them, offering a principled way to interpret cases where experiential coherence and inferential success come apart.

Much work remains, particularly in the mathematical formalisation of the awareness field and its projection structure. However, the central claim advanced here does not depend on the success of any particular formalism. It rests on a simple recognition: coherent awareness is invariant across all change, in the sense that intelligibility requires the preservation of unity, temporal continuity, and reflexive availability across transformation; nothing intelligible can appear without the maintenance of such coherence. Making this invariant explicit allows the variational principles of science to be seen not as metaphysically exhaustive, but as precisely what they have always been-lawful descriptions of coherence under constraint.

In this sense, the AFT does not seek to replace existing theories. It explains why they work, where they apply, and why they stop where they must. By restoring coherence of awareness to its rightful place as a condition of possibility, the framework offers a unified and conceptually stable foundation for understanding inference, physics, and experience within a single variational architecture.

## Data Availability

No new data were created or analysed in this study. Data sharing is not applicable to this article.

## Appendix A. Invariance, Projection, and the Condition of Appearance

A difficulty in consciousness science and fundamental physics concerns the relationship between invariant structure and its observable manifestations. Scientific theories successfully describe regularities, dynamics, and symmetries within experience, yet they remain systematically silent about the condition under which anything appears at all. The AFT (AFT) argues that this silence is not a failure of science, but a structural consequence of projection: invariant generators do not appear within their projections.

This appendix offers a conceptual illustration of that claim by bringing together three elements: (i) Plato’s allegory of the cave, (ii) the variational structure articulated in the main text, and (iii) a simple mathematical analogue based on Euler’s unit circle. The aim is not to introduce a new argument, but to make vivid the logical structure already operative within the theory.

### Appendix A.1. Projection and the Allegory of the Cave

In Plato’s allegory of the cave, prisoners observe shadows cast on a wall and mistake them for reality. The crucial point is not merely that the shadows are incomplete, but that the source of the shadows cannot appear within the same representational register. The fire and the objects behind it are not hidden shadows; they belong to a different explanatory level altogether.

In the AFT, third-person scientific descriptions—physical dynamics, neural activity, inferential processes—occupy a role structurally analogous to the shadows. They are lawful, structured, and indispensable. However, they are projections: partial descriptions of a deeper invariant condition that cannot itself appear as an object within those descriptions.

The Coherence Principle (δA=0) plays a role analogous to the “sun” in Plato’s analogy—not as an entity, but as a condition of intelligibility. It is not something that could appear within physics or inference, because physics and inference already presuppose it. To demand that awareness appear within third-person science is therefore to demand that a generator show up as one of its own projections.

### Appendix A.2. Euler’s Unit Circle as a Toy Model of Projection

This logic can be illustrated using a simple mathematical structure. Consider Euler’s identity:

$$e^{i\theta }=\cos\theta +i\sin\theta$$

Here, the unit complex exponential represents a rotation of invariant magnitude. As the parameter θ varies, the system undergoes continuous change, yet the modulus remains fixed at unity. The invariance is real, but it does not appear directly in either the cosine or sine components.

The real and imaginary parts are projections of the same underlying rotational structure onto orthogonal axes. Each projection is lawful, continuous, and measurable. Each can be studied independently. Yet neither projection contains the invariant generator itself. No refinement of the cosine function will ever reveal that the motion is rotational; that fact belongs to the generating structure, not to its projections.

This analogy is not intended to suggest that awareness is mathematically identical to rotation. Rather, it illustrates a general structural relation between invariance and projection. In the AFT, the correspondence is as follows: the Coherence Principle (δA=0) plays the role of an invariant generator; the free-energy principle (δF=0) and stationary-action principles (δS=0) correspond to distinct projections under different constraints. Inferential and physical laws are therefore not false or illusory, but incomplete in a principled and mathematically natural way.

### Appendix A.3. Projections as Inference and Physics

From this perspective, Active Inference and physical law are coordinated projections of the same invariant coherence condition. Free-energy minimisation describes how locally bounded systems—defined by Markov blankets—maintain coherence under uncertainty. It is a projection of coherence into probabilistic belief space, where trajectories are constrained by expectations, precision, and action.

Stationary-action physics describes how coherence propagates when phenomenological structure is abstracted away entirely. It is a projection into relational state space, yielding stable laws that are insensitive to perspective or experience. Neither projection contains awareness itself, just as neither sine nor cosine contains rotation. Both, however, are governed by the same invariant generator.

### Appendix A.4. Markov-Blanketed Systems as Local Trajectories

Within this framework, what is ordinarily called an organism or agent corresponds to a locally stabilised trajectory within the awareness manifold. A Markov blanket defines a local coordinate system—a way of partitioning internal and external states—allowing that region to infer itself and its environment.

At any moment, a Markov-blanketed system occupies a point in its constrained state manifold. The tangent space at that point encodes the admissible directions of change: bodily movement, perception, affect, cognition, and action. Active inference describes how the system follows a coherence-preserving path through this space by reducing free energy. Across these transformations, appearances change continuously and worlds unfold, while the invariant condition—the coherence of appearing itself—remains unaffected. Becoming unfolds within being; being is not threatened by becoming. In this sense, the classical tension between Parmenidean permanence and Heraclitean flux appears not as a contradiction but as a difference of level: invariance at the level of coherence, transformation at the level of trajectories.

### Appendix A.5. What This Clarifies

This clarifies several points that often remain puzzling. First, it explains why awareness does not appear in third-person science: awareness is not an object within the domain science describes, but the invariant condition under which description occurs. Second, it clarifies why increasing descriptive complexity cannot resolve the hard problem of consciousness, since no projection, however detailed, can contain its generator. Third, it preserves the full validity of physics and inference, which remain lawful, necessary, and powerful precisely because they are projections rather than eliminations. Finally, it shows why the Coherence Principle δA=0 is not speculative, but articulates what is already logically unavoidable once the structure of explanation itself is made explicit.
